# Propofol versus placebo (with rescue with ketamine) before less invasive surfactant administration: study protocol for a multicenter, double-blind, placebo controlled trial (PROLISA)

**DOI:** 10.1186/s12887-020-02112-x

**Published:** 2020-05-08

**Authors:** Marie Chevallier, Xavier Durrmeyer, Anne Ego, Thierry Debillon, The PROLISA Study Group, Alain Beuchee, Laura Bourgoin, Aurélie Desenfants, Amélie Durandy, Cyril Flamant, Géraldine Gascoin, Ghida Ghostine, Johanna Parra, Laure Ponthier, Jean-Michel Roué

**Affiliations:** 1 UMR 5525 ThEMAS, CNRS, TIMC-IMAG, Grenoble Alps University, Grenoble, France; 2 Neonatal Intensive Care Unit, Grenoble Alps University Hospital, Grenoble, France; 3 Neonatal Intensive Care Unit, Centre Hospitalier Intercommunal de Créteil, Créteil, France; 4 Université Paris Est, IMRB- GRC GEMINI, Créteil, France; 5 Inserm, U1153, Obstetrical, Perinatal and Pediatric Epidemiology Team, Epidemiology and Biostatistics Sorbonne, Paris Descartes University, Paris, France

**Keywords:** Less invasive surfactant administration, Sedation, Propofol, Ketamine, Randomized study

## Abstract

**Background:**

One major limitation for less invasive surfactant administration (LISA) is the difficulty in providing sedation before this procedure and the competitive risk of respiratory depression versus avoidance of intubation for most sedative or analgesic drugs used in this context.

The objective of this study is to compare the need for mechanical ventilation within 72 h of life following premedication with propofol, versus placebo (rescue with ketamine), for the LISA procedure in preterm neonates born before 32 weeks gestational age (wGA).

**Methods:**

ProLISA is a phase III, non-inferiority, multicenter, double blind, randomized, placebo controlled trial designed according to the SPIRIT Statement. Neonates born before 32 wGA in 12 geographically dispersed Neonatal Intensive Care Units in France needing surfactant will be included from September 2019 to September 2022. A sample of 542 patients is needed. The neonate is randomized to the intervention (propofol) or control placebo group. Open label rescue treatment with ketamine is possible in both groups if FANS (Faceless Acute Neonatal pain Scale) is ≥6. To guide drug administration, FANS is scored before attempting laryngoscopy. Once an adequate score has been obtained, LISA is performed according to a standardized protocol. The primary outcome is the need for mechanical ventilation within 72 h of life. Secondary outcomes are tolerance of the procedure, pain evaluation, hemodynamic and neurologic parameters after the intervention, morbidities before discharge and neurodevelopmental assessment at 2 years of age.

**Discussion:**

This paper describes the first multicenter, double-blind, randomized, placebo-controlled trial on this topic and will provide crucial information to support implementation of the LISA procedure.

**Trial registration:**

ClinicalTrials.gov: NCT04016246. Registered 06 June 2019, N°EUDRACT: 2018–002876-41.

## Background

Respiratory Distress Syndrome (RDS) affects 85% of preterm babies born before 32 weeks gestational age (wGA). Currently, the strategy to manage RDS in neonates who do not require intubation in the delivery room includes non-invasive nasal ventilation, early rescue treatment with animal derived surfactant, and the limitation of mechanical ventilation (MV). In fact, MV is one of the main risk factors of bronchopulmonary dysplasia (BPD) because it leads to chronic inflammation [1–3]. A reduction in the duration of MV is also one of the ways to limit the risk of BPD [4]. European guidelines from 2019 state that “LISA [less invasive surfactant administration] is the preferred mode of surfactant administration for spontaneously breathing babies on continuous positive airway pressure (CPAP), provided that clinicians are experienced with this technique” [5]. In the LISA procedure, the operator inserts a thin tube to administer intra-tracheal surfactant to spontaneously breathing preterm infants receiving non-invasive ventilation [6–15]. After surfactant administration, the catheter is immediately removed and non-invasive ventilation is continued [6]. The main advantage of this strategy is to avoid ventilator-induced lung injury. Two meta-analyses reported a 30% decrease in MV within the first 72 h and in bronchopulmonary dysplasia (BPD) at 36 weeks with the use of LISA [16, 17]. These meta-analyses pooled and studied the data of several randomized trials about LISA versus other strategies. One of the barriers to the wider implementation of the technique is the absence of consensus on premedication [18]. In fact, most studies were performed without sedation or with sedation at the discretion of the clinicians [19].

Thus an ideal drug for this context should have fast onset, short action, rapid offset, provide good sedation and analgesia with the least possible adverse effects and especially little impact on respiratory drive.

Currently there is no international and national consensus concerning specific premedication prior to the LISA procedure. Unfortunately the most interesting drugs induce respiratory depression and could lead to intubation, which is exactly what the LISA procedure tries to avoid. Propofol, ketamine and fentanyl have been reported in the literature as premedication for the LISA procedure [11, 13, 14, 20]. In existing studies using premedication, the overall population had longer gestational ages but slightly higher MV rates as compared to studies without premedication.

Propofol might be of interest due to its short action, quick preparation, rapid onset and sedative properties [21]. It has been shown to be a suitable sedative for non-emergent neonatal endotracheal intubation with good tolerance and the possibility of titration [22, 23]. An observational study reported a higher level of comfort (ComfortNeo scale) when using premedication with 1 mg/kg propofol for the LISA procedure compared to a historical non-sedated control group [11]. An unmasked, single-center randomized, controlled trial of 1 mg/kg propofol versus no premedication during LISA found a significant decrease in the pain score (ComfortNeo) but more frequent desaturations in the propofol group. No significant difference was observed for the rate of intubation, but the study might have been underpowered for this outcome [20].

Ketamine has rapid onset, a short duration of action, good analgesic and amnesic properties and maintains cardiovascular and respiratory stability [24]. An observational study reported the use of ketamine titrated by 0.5 mg/kg increments in neonates prior to the LISA procedure [13]. The intubation rate was 24% during the procedure and 41% within 72 h, underlining the difficulty to assess the depth of sedation. However, the majority of infants had faceless acute neonatal pain scores (FANS) below the threshold for pain. In another observational study on neonatal intubation in the delivery room, ketamine was associated with decreased pain scores, as compared to no premedication [25].

Briefly, propofol and ketamine seem to be effective for reducing pain scores, with acceptable tolerance for the LISA procedure, although propofol seems to be easier to titrate in this population.

Since 1990, studies have demonstrated that direct laryngoscopy is a painful and stressful procedure [26] that is why performed LISA without premedication is hard to discuss. It may cause bradycardia and intracranial hypertension when it is performed without sedation [27, 28]. For this reason, several academic bodies have recommended the use of premedication before neonatal intubation, except in life-threatening situations [26, 29]. Despite this evidence, a Scandinavian survey showed that only half of responding NICUs always used premedication before a LISA procedure, although it requires a laryngoscopy similar to that required by endotracheal intubation [30]. The clinicians’ major concern about using premedication before LISA is the opposing goals of obtaining efficient sedation/analgesia while maintaining respiratory drive. For these reasons, this study assesses the impact of sedation during LISA on the rate of MV. Our hypothesis is that using premedication with propofol in a standardized protocol will not dramatically increase the need for MV within 72 h.

The objective is to conduct a non-inferiority study to compare the need for MV within 72 h of life following premedication with propofol or placebo before the LISA procedure in preterm babies < 32 wGA.

## Methods/design

### Study setting and design

PROLISA is a phase III, non-inferiority, multicenter, double blind, randomized, controlled trial comparing propofol vs placebo. The trial involves 12 geographically dispersed NICUs in France. These neonatal units regularly participate in health research and have a varied case mix of infants. All centers already use the LISA procedure as a standard of care. In each center, a PROLISA referent neonatologist has been appointed.

### Study sample and recruitment

Infants born before 32 wGA are included as soon as possible after birth from September 2019 to September 2022, once they meet the inclusion criteria:

- presenting a RDS in the first 48 h of life and treated by non-invasive ventilation requiring surfactant with the following FiO_2_ to obtain a pulse oxymetry (SpO_2_) between 88 and 95%
  - FiO_2_ ≥ 30% for a duration ≥10 min for infants born between 28 and 31 wGA
  - FIO_2_ ≥ 25% for a duration ≥10 min for infants born < 28 wGA;
- available intravenous line (peripheral, umbilical or central catheter);
- covered by the French Social Security;
- signed parental informed consent.

Non-inclusion criteria are:

- congenital and/or major malformations, including upper airway malformations;
- FiO_2_ > 60% at the time of inclusion;
- Silverman score > 6;
- Contra-indication to the use of propofol:
  - low mean arterial blood pressure at 2 successive measurements (< gestational age expressed in weeks persisting after one volume expansion),
  - use of inotropic medication to maintain a normal blood pressure.
  - use of sedative or analgesic drugs (except paracetamol and ibuprofen) in the previous 24 h
  - coma, seizures, areactivity at neurological examination

### Interventions

#### Management before procedure

After verifying the neonate’s eligibility, obtaining the mother’s, and ideally both parents’ consent, the newborn is randomized to the Propofol or placebo arm. For the intervention, the newborn is prepared as per usual practice for tracheal intubation, whether admitted to the NICU or resuscitated in the delivery room, equipped with cardio-respiratory monitors, SpO_2_ monitoring (sensor placed on right hand). The equipment for the LISA procedure and for tracheal intubation (endotracheal tube, laryngoscope and Magill Forceps) is prepared. Non-invasive respiratory support (with devices routinely used in the centre) is maintained throughout the procedure using Non Invasive Positive Pressure Ventilation (NIPPV) or CPAP with Positive End Expiratory Pressure (PEEP) ≥ 4 cm H_2_0, Positive Inspiratory Pressure (PIP) ≥6 cm H_2_O, and respiratory frequency at the discretion of the clinicians. However, we propose a respiratory rate around 30/ min. NIPPV devices must have including variable flow and constant flow generators.

In the two groups, when the intervention team is ready, a solution of atropine, caffeine and oral sugar solution 30% or 24% is given drop, wise orally using a syringe before the LISA procedure. The posology of drugs are chosen according to the protocol routinely in use locally (around 10 μg/kg for atropine, 20 mg/kg for caffeine and 5–10 drops for sucrose). If LISA is performed in delivery room, caffeine should be given at soon as possible after procedure. It is common practice in France for atropine to be used before any intubation procedure. The aim of the atropine administration is to reduce the risk of bradycardia associated with an exacerbation of vagal tone. Sucrose is given as an analgesic agent [31] and caffeine is used to avoid central apnea [32].

#### Intervention: sedation in both experimental and control groups

The administration of propofol (experimental group) or placebo (control group) is performed by titration: (0.5 mg/kg per dose of propofol or a similar volume of placebo (20% Medialipide).

**Fig. 1:**
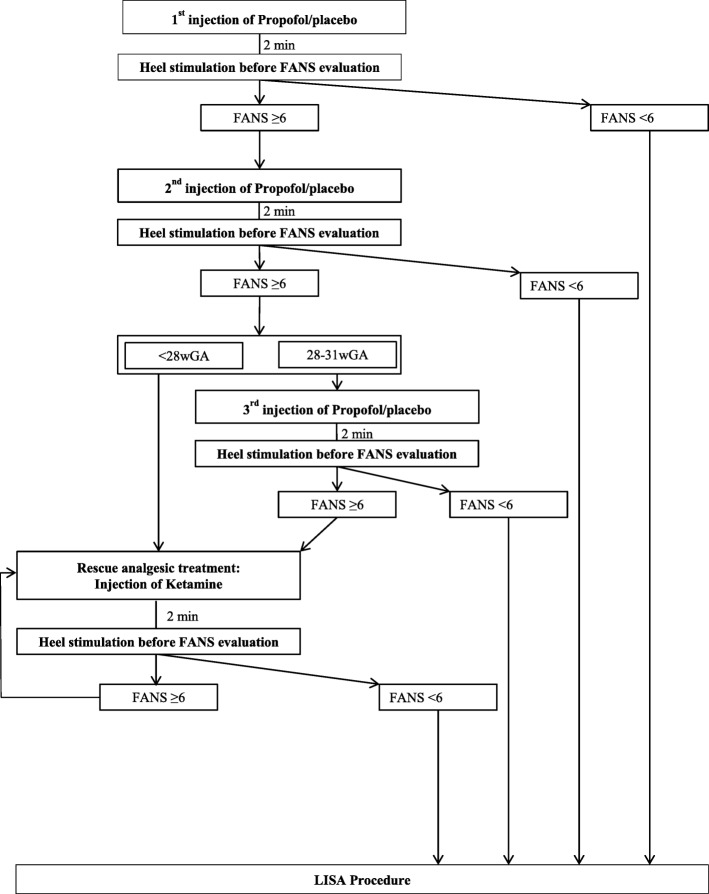
Intervention scheme

Within 2 min of injection of the first dose, a FANS pain score is rapidly evaluated after firmly rubbing the neonate’s heel; according to a previously published intubation readiness score [33] **(**Fig. 1**)**: The FANS scale (/10) allows a painful procedure to be evaluated, whether the face is visible or not.

- If the FANS score is ≥6, a second dose of 0.5 mg/kg is injected. After two (< 28 wGA) or 3 (28–31 wGA) administrations of the drug (or placebo), if sufficient comfort is not achieved (FANS ≥6) an open label rescue treatment is given: ketamine at 0.5 mg/kg. One or two injections are recommended, but in some cases further doses of ketamine doses might be needed according to the discretion of the clinician. The latter should be an exceptional case, and 1 or 2 doses should be sufficient in most cases. This open-label treatment with ketamine is consistent with expert recommendations based on trials evaluating pain treatment in neonates [34].
- If the FANS score is < 6, no further injection of propofol (or placebo) is given.

#### The LISA procedure

An aspiration probe (CH6) or the LISAcath® (Catheter for oral endotracheal instillation, CHIESI SAS, Bois Colombes, France) is used. The type of probe is left to the choice of each investigator. If an aspiration probe is used, it is marked to indicate the desired depth of insertion (6 cm + weight if the procedure is performed through the nose, 7 cm + weight if the procedure is performed through the mouth with a nasal mask left in place during the procedure). Direct laryngoscopy or indirect laryngoscopy (with videolaryngoscopy), according to local practice, is performed with the probe inserted to beyond the vocal cords at the required depth, and held in position at the lips. Once the probe is correctly positioned, surfactant is slowly infused at a dose specified by the local protocol. At the end of the administration, the probe is immediately removed. The newborn continuously remains on NIPPV throughout the procedure, since in previous trials using propofol for LISA, NIPPV was found to be required continuously [11, 20].

If the newborn develops transient apnoea, inflations providing additional positive pressure are given using the respiratory support (NIPPV), an Ambu® bag or a T-piece resuscitator device (Neopuff®).

The previous experience of the laryngoscopy operators in the technique is recorded (number of previous LISA procedures). Junior operators are allowed to perform the procedure supervised by a senior operator ready to take over if required.

### Outcomes

#### Primary outcome

The primary outcome is the need for MV from the LISA procedure onwards up to 72 h of life indicated according to standardized criteria:

- repeated and severe apnea (defined by the American Academy of Pediatrics Guidelines) with bradycardia and/or low oxygen saturation) [35];
- high FiO_2_ (according to local practices) justifying intubation for a second administration of surfactant and MV;
- respiratory complications such as pneumothorax, with an aggravation of the RDS;
- any other cause where the clinician judges MV to be necessary: The motive for the respiratory assistance is indicated in the Case Report Form (CRF). All intubations for MV is recorded.

#### Secondary outcomes

- The FANS score is collected before, during and after the LISA procedure in both groups (propofol and placebo).
- Ketamine administration (number of injection(s), dose for rescue (if FANS≥6) is also recorded.
- Per procedure events are recorded including the number of laryngoscopies needed to perform LISA, the evolution of cardiorespiratory parameters from baseline to 1, 3, 5, 15, 30, 60 and 120 min after the first drug injection: heart rate, respiratory rate, pulse oxymetry, blood pressure, FiO_2_, ventilatory mode, inspiratory and end-expiratory ventilation pressures, transcutaneous pCO_2_, the presence of apnoea requiring bag mask ventilation or additional nasal pressure with NIPPV and the emergency intubation for severe apnea after the drug injection before the LISA procedure.
- After the procedure: the clinician who successfully performed the LISA rates his/her performance during the laryngoscopy (especially the facility of exposure of the glottis) according to the Viby-Mogensen scale [33, 36]. This score based on 5 items (scored from 0 to 4), explores the facility to expose the larynx and the infant’s behavior [36].

**Fig. 2:**
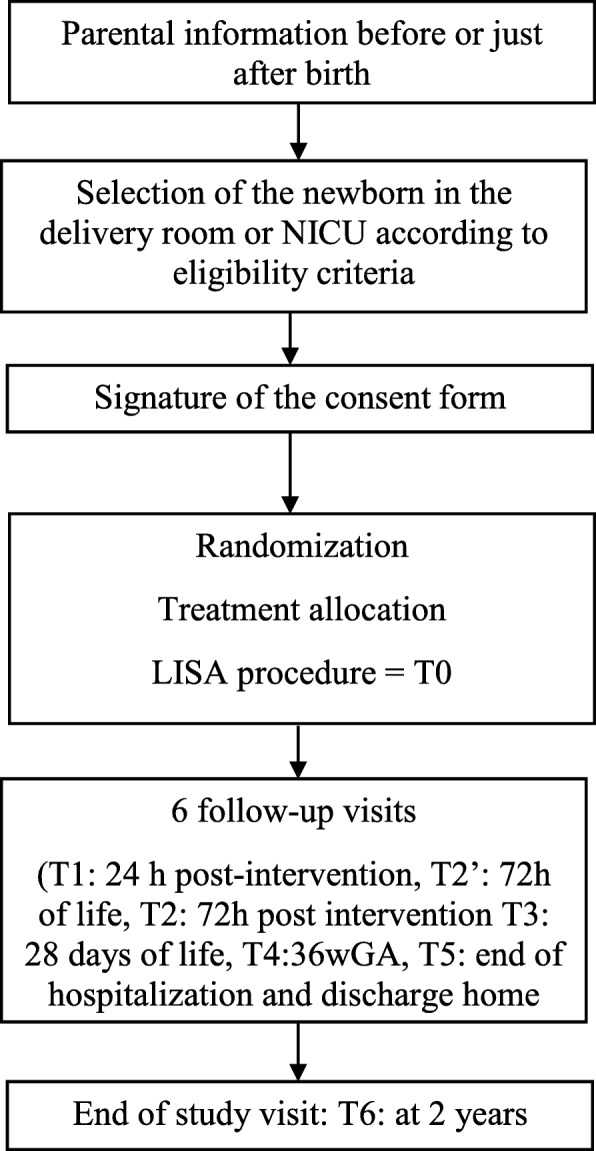
Time frame

Others items related to the tolerance of the procedure such as cardiorespiratory and neurological parameters are collected at 24 h and 72 h after the intervention:

- in-hospital neonatal morbidity and mortality: pneumothorax, necrotizing enterocolitis according to Bell stage [37], proven early and late onset sepsis (respectively defined as positive CSF or blood culture before< and after> 72 h of life), retinopathy of prematurity according to international classification [38], cystic periventricular leukomalacia or grade 3 or 4 intraventricular hemorrhage according to the Papile classification [39], surgical treatment of patent ductus arteriosus, duration of cumulated MV, duration of cumulated non-invasive ventilation, any intubation, death at 36 wGA and in-hospital mortality.
- Data at 2 years of age will be collected: Standard pediatric examination, Age and stage questionnaire (ASQ) [40] completed by the parents, Gross Motor Function Classification Score [41] in cases of motor impairment, and any visual and/or hearing disabilities detected during the first 2 years of life **(**Fig. 2**)**.

### Consent

Considering the emergency context of most situations, anticipated informed consent is sought from mothers, and ideally both parents, as soon as possible after the admission of the mother to the delivery room or at admission of the neonate to the NICU. It is possible to propose the study before birth if the mother is previously hospitalized in the obstetrical unit. Initially a single parent’s signature is sufficient to include the newborn in the study. The recovery of the second parent’s signature is sought within 48 h of the neonate’s inclusion.

### Sample size

The study has a non-inferiority objective for MV within 72 h. The type I error is set at 5% and the power at 80%. We expect a 30% rate of MV in the control group which corresponds to the percentage of intubated patients 72 h after the LISA procedure in the AMV study [6]. The non-inferiority margin considered as clinically acceptable is set at 10%. In this setting, a total of 542 neonates will be recruited (271 per group).

### Recruitment and allocation

As the total number of patients needed is 542 the study is multicentric, with the involvement of 12 motivated neonatal units in university hospitals over 3 years. Each center’s estimate for recruitment was based on the usual level of activity of the NICU.

A predefined randomization list with a 1:1 ratio, obtained using a random number generator, with a random size of blocks from 2 to 6, was established. The randomization was stratified by center and by class of GA (< 28, 28–32 wGA). Clinical staff are unaware of the allocation sequence, which is communicated only to the Grenoble hospital pharmacy.

### Blinding

Drugs are delivered by the hospital pharmacy as required in sequentially numbered containers. Medialipide® is used in the control group to ensure that the 2 arms are indistinguishable. Medialipides® is an emulsion of medium and long triglycerides based derived from soya oil and has the same appearance as propofol [20, 22].

### Data collection, management, analysis, auditing

A learning phase involving junior and senior neonatologists has been planned in all centers to ensure the homogeneity of practice including sedation/placebo injection, monitoring during the procedure, and rescue analgesia. Training is supported by videotapes illustrating the LISA procedure and relayed by the referent neonatologist in each participating center. It also includes examples for scoring FANS. During the LISA procedure one or two observers not involved in the procedure are assigned to the collection of data (FANS, cardiorespiratory parameters etc.). Physiological parameters are extracted from the monitors to serve as source data. In each center, one clinical research assistant is in charge of entering all the data in an electronic CRF, registered with a secure interface managed by the Grenoble Alps University Hospital.

During the clinical research or after its termination, the collected data will be anonymized before being communicated to the sponsor by the investigators (or any other specialist).

A clinical research assistant mandated by the sponsor regularly visits the study centers: while the study is being set-up, one or more times during the course of the research depending on the rate of inclusions and at the end of the research. The elements to be monitored during these visits and the frequency of these visits have been defined prior to the start of the study in collaboration with the investigation team and according to an assessment of the risk level of the study. All visits are the object of a written report and a copy is forwarded to the principal investigator.

Any written or oral communication of research results must receive the prior approval of the principal/coordinating investigator and, of any committee established for the research.

### Harms

All the infant’s vital signs are monitored throughout and after the intervention and any significant adverse event, adverse drug reaction (hypotension, severe apnea, emergency intubation after drug injection) or unexpected adverse drug reaction (according strict definitions) are reported by the investigator to the sponsor’s service in charge of safety and regardless of any causal relationship concerning the drug under investigation or the research. In case of suspected adverse reactions and if the knowledge of the treatment administered allows a specific management, unblinding can be requested by the investigator even in case of emergency. Unblinding may be done 24/24 h by the Pharmacy. A pharmacologist, and two neonatologists who are independent of the study compose the independent monitoring committee. They must assess whether there is any causal link between the SAE and the experimental drug or procedure. This committee meets at the request of the sponsor or principal investigator, after 50 inclusions, annually or after any intermediary analysis. They can purpose four types of action: continue following the protocol, asking for a complementary analysis, asking for a protocol amendment, stopping the study (for patient security reasons).

### Statistical methods

Considering that an intention to treat analysis may not be appropriate for non-inferiority trials [42] a per-protocol analysis, including all patients who satisfactorily complied with the assigned treatment and who had no major protocol violations, will be conducted. An intention to treat analysis will be carried out subsequently to confirm the results of the per-protocol analysis. The threshold *p* < 0.05 will be considered to define the significance of the statistical tests performed with Stata MP15. Blinding will be maintained during the statistical analysis.

#### Primary outcome

The need for MV within 72 h will be assessed in each group. This trial aims to show that the experimental arm with sedation is no less effective than the control arm with placebo, and we made the hypothesis that the rate of MV within 72 h of life will not exceed 40% with sedation, compared to 30% in the control group [6], corresponding to an equivalence margin of 10%. If the upper limit of the one-sided 95% confidence interval for the treatment difference by the experimental treatment is less than this equivalence margin, then we will retain the non-inferiority of the experimental strategy at a 5% significance level. If non-inferiority is rejected, the rates of MV up to 72 h life will be compared using a conventional superiority test.

#### Secondary outcomes

The analysis of the primary outcome will be repeated by class of GA (< 28, 28-31wGA). Means and standard deviations (or median and 25th and 75th percentile) of FANS, cardio-respiratory parameters, number of laryngoscopies, number of apnoeas, clinician’s satisfaction, neurodevelopmental outcomes at 2 years (ASQ, GMFCS, visual and hearing function), will be calculated and compared using the Student test (or Mann-Whitney test).

Numbers and frequencies of ketamine administration for rescue and emergency intubation, pneumothorax within the first 72 h, BPD at 28 days and 36wGA, other outcomes of neonatal morbidity and in-hospital mortality will be calculated, and compared using the Chi2 test (or Fischer exact test).

#### Interim analysis

An interim analysis of the primary endpoint will be performed once half the planned number of newborns have been included. This interim analysis will aim to confirm patient safety and help decide on the continuation or discontinuation of the study (stopping for toxicity or stop for futility). To maintain an overall level of 5% in the final analysis, the interim analysis will be performed with a threshold of 0.1%. The results of the interim analysis will be discussed by the steering committee and safety committee who may propose amendments to the statistical analysis plan.

#### Missing data

Considering the design and that endpoints are mostly recorded during hospitalization, a high rate of missing data is unlikely. Nevertheless, in case of rates between 5 and 20%, missing data will be replaced. The replacement of missing data will either be according to an analysis strategy for the worst case scenario, or by multiple imputation. If multiple imputation is used, 5 imputations will be made, using a logistic regression model taking into account the main factors related to the outcome.

### Research ethics approval

The study n°2018–002876-41 (EudraCT) was approved by the French research ethics committee on March 3, 2019 and by the French drug administration, on October 5, 2018. If there is a need for protocol amendments, the principal investigator has to inform the different investigators, trial participants, the promotor and the amendments should be presented to the French research ethic committee for approval.

## Discussion

In Europe the widespread use of the LISA technique and the known deleterious effects of laryngoscopy in unsedated neonates prompted researchers to evaluate the feasibility of premedication for the LISA procedure. In the study by Heiring et al., of 73 North European NICUs performing the LISA procedure 78% declared using medication with a large variety of drugs [30]. Currently the most studied drug is propofol [11, 12, 20] with promising preliminary results from a single center, unmasked, randomized controlled trial versus no premedication [20]. Moreover, we did not choose fentanyl as a rescue treatment due to the major risk of chest wall rigidity and thus intubation [14, 43].

Our protocol has two limitations: the open label treatment with ketamine in both groups and the multiple possible indications for intubation. Concerning the first limitation, a “no sedation” control group for a painful procedure (LISA) seems to be unethical. Therefore we planned rescue treatment using ketamine as suggested by Berde et al. for pediatric analgesic trials [34]. This design is not perfect to evaluate a medication, but it seems to be best for the included neonate. Ketamine rescue and the number of doses required will be an interesting factor to compare between the two groups. It may reflect the potential pain management across the both group.

Regarding the second limitation, we considered that the use of mechanical ventilation after a LISA procedure should be similar to real-life practice. Although indications for MV were not strictly standardized, the results obtained will have reinforced generalisability. This approach was also adopted in the initial AMV trial [6] and has been retained in the present study. If our hypothesis of non-inferiority and safety is verified, it provides a strong argument for performing LISA with propofol sedation. This could have an impact on the generalization of LISA, which may have real benefits for infants with bronchopulmonary dysplasia.

This trial will be the first multicenter, double blind, randomised, placebo controlled trial evaluating premedication before LISA with an expected number of participants of 542. In addition it will examine the neurodevelopmental outcome at 2 years of age of preterm infants who received a few doses of propofol or ketamine shortly after birth [22, 44].

Our study should provide robust data on premedication use before a routine LISA procedure for clinicians studying tolerance, efficacy and adverse events. Furthermore, it may contribute to improving the comfort and pain management of neonates during the LISA procedure.

## Data Availability

The data recorded during this research are subject to computer processing by the clinical research center, Grenoble Alps University Hospital in accordance with Law No. 78–17 of 6 January 1978 on computers, files and freedoms amended by Law 2004–801 of August 6, 2004.

## Abbreviations

BDP: Bronchopulmonary dysplasia
CRF: Case report form
FANS: Face acute neonatal scale
LISA: Less invasive surfactant administration
MV: Mechanical ventilation
NICU: Neonatal intensive care unit
NIPPV: Non-invasive positive pressure ventilation
RDS: Respiratory distress syndrome
wGA: weeks of gestational age
